# Excess Risk of Lung Cancer Among Agriculture and Construction Workers in Indonesia

**DOI:** 10.5334/aogh.3155

**Published:** 2021-01-06

**Authors:** Anna Suraya, Dennis Nowak, Astrid Widajati Sulistomo, Aziza Ghanie Icksan, Ursula Berger, Elisna Syahruddin, Stephan Bose-O’Reilly

**Affiliations:** 1 CIHLMU Center for International Health, University Hospital, LMU Munich, Germany; 2 Occupational Health and Safety Program, Universitas Binawan, Jakarta, Indonesia; 3 Institute and Outpatient Clinic for Occupational, Social and Environmental Medicine, LMU University Hospital, Munich, Germany; 4 Comprehensive Pneumology Center Munich (CPC-M), Member, German Center for Lung Research (DZL), Germany; 5 Occupational Medicine Specialist, Universitas Indonesia Academic Hospital, Jakarta, Indonesia; 6 Department of Radiology, Persahabatan Hospital, National Respiratory Referral Hospital, Jakarta, Indonesia; 7 Faculty of Medicine UPN Veteran, Jakarta, Indonesia; 8 IBE – Institute for Medical Information Processing, Biometry and Epidemiology, LMU Munich, Germany; 9 Division of Thoracic Oncology Department of Pulmonology Faculty of Medicine, Universitas Indonesia Persahabatan hospital, Jakarta, Indonesia; 10 Institute of Public Health, Medical Decision Making and Health Technology Assessment, Department of Public Health, Health Services Research and Health Technology Assessment, UMIT–Private University for Health Sciences, Medical Informatics and Technology, Austria

## Abstract

**Background:**

In Indonesia, many occupations and industries involve a variety of hazardous and toxic materials. The ILO estimates that about 21.1% of the tracheal, bronchial, and lung cancer deaths among men were attributable to workplace hazardous substances. This study investigated the relationship between occupations or workplace exposure and the risk of lung cancer in the country. The results will help determine how Indonesia can best mitigate the risk for its workers.

**Objectives:**

This case-control study utilizes the Indonesian Standard of Industrial Classification (IndSIC) 2015 with the aim of exploring the risk of lung cancer among Indonesian workers.

**Methods:**

The study included patients aged 35 years old or older receiving thoracic CT at the radiology department of Persahabatan Hospital. The cases were histological-confirmed primary lung cancers, while the controls were negative thoracic CT scan for lung cancer. The subjects’ job titles and industries were classified according to IndSIC 2015 and blind to the patient’s grouping as a case or control. Logistic regression was used to determine the odds ratios for lung cancer among all sections and some divisions or groups of IndSIC 2015.

**Findings:**

The mean age was 58.1 (±10.23) years for lung cancer patients and 54.5 (±10.23) years for controls. The majority of subjects (19.6%) worked in Section G (Wholesale and retail trade; repair of motor vehicles and motorcycle). After adjusting for age, gender, level of education, and smoking habit, the risk of lung cancer was nearly three-times higher (OR = 2.8, 95% CI = 1.11–7.02) in workers of Division A01 (crop, animal production, and hunting) and two-times higher (OR = 1.9, 95% CI = 1.05–3.46) in workers of Section F (construction) compared to the workers in other sections or divisions.

**Conclusions:**

The excess risk of lung cancer among certain categories of workers confirms the need for improved policy, monitoring, and control of occupational exposure for primary cancer prevention and workers’ compensation purposes.

## INTRODUCTION

The International Agency for Research on Cancer has listed 19 substances with sufficient evidence for lung carcinogenicity in humans (Group 1) [1,2]. Many occupations and industries in Indonesia involve a variety of carcinogenic materials, such as asbestos and silica in construction and renovation work, welding fumes in steel processing industries, and diesel exhaust for truck drivers and operators of machine engines [2,3]. Epidemiological studies have reported the increased risk of lung cancer development in several occupations [4,5,6]. According to the global estimates of occupational accidents and work-related illnesses reported in 2017, about 21.1% of the tracheal, bronchial, and lung cancer deaths among men were attributable to workplace hazardous substances including dust, vapors, and fumes [7].

The number of lung cancer incidents in Indonesia has increased over time and occurs at a younger age compared to other countries [8]. In 2018, the WHO reported 30,023 new cases of lung cancer in Indonesia and 26,095 deaths, making up around 2.6% of Indonesia’s total deaths [9]. Unfortunately, a limited number of studies have been conducted regarding the relationship between occupations or workplace exposure and the risk of lung cancer in the country [10,11]. Until 2020, occupational lung cancer had not been reported to the Indonesian government, and the occupational risks for lung cancer among Indonesian workers remained unclear [12].

Approaching the relationship between workplace exposure and lung cancer is very challenging. The most notable intricacies in recognizing the relationship is the long latency period of lung cancer and strong confounding factors, like smoking. Indonesia also lacks data on occupational exposure. This further complicates identifying the relationship between occupational agents and lung cancer.

The International Standard Industrial Classification (ISIC) is the system established by the United Nations to classify economic activities [13]. In 1977, Indonesia adopted a system called the “Indonesian Standard Industrial Classification” (IndSIC) or “Klasifikasi Baku Lapangan Usaha Indonesia” (KBLI) [14]. The extensive use of the classification has made it a tool for when studying the economic phenomena as well as employment, health data, and other matters [13,15]. Amid limited occupational exposure data, IndSIC can be used as a proxy for different exposures for each classification [16]. When implementing the ISIC, many studies have successfully discovered the risk of lung cancer among workers in many countries [3,17].

This study set out to determine the association between occupational exposure proxied by IndSIC and the risk of developing lung cancer among Indonesian workers. Having health information based on the economic activities’ classification may bring new insight into occupational health research and cancer prevention programs in Indonesia.

## MATERIALS AND METHODS

We conducted this study with the review and approval of the Ethical Committee of Persahabatan Hospital, Indonesia, (number 18/KEPK-RSUPP/03/2018) and the Ethical Committee at the medical facility, Ludwig-Maximilians University in Munich, Germany (number 18–632). All subjects signed an informed consent form after receiving the participants’ information on all aspects of the study.

### STUDY POPULATION

For 17 months between May 2018 and September 2019, we performed a case-control study at the National Respiratory Hospital in Jakarta, Indonesia. The recruitment of cases and controls in this study followed a protocol similar to what was utilized in a previously published study by the same authors on asbestos-related lung cancer in a hospital-based case-control study in Indonesia [18]. The study population consisted of all patients aged 35 years old or older who received a thoracic computerized tomography (CT) scan for a range of indications including lung infection, mediastinal mass, lung nodule or mass, trauma, and evaluation of pleural diseases. The cut off age of the subjects, 35 years, was chosen based on the assumption that the youngest working-age in Indonesia is 15 years old and the average latency period for developing lung cancer is around 20 years; therefore, it is estimated that the youngest occupational lung cancer develops around the age of 35 years [19,20].

The cases were primary lung cancers that were confirmed by histology, and the controls were recruited from the same as those who had the thoracic CT scan but returned images with no evidence of lung cancer. The CT scans were interpreted by a thoracic radiologist, and the histological information was obtained from the hospital’s pathology department.

Trained interviewers carried out the interviews using a standardized questionnaire adapted from Cancer Research UK [21] and were translated by a certified translator into Bahasa, Indonesia. We obtained the information on demographic data, smoking habits, and lifetime occupational history including industry category, job titles, and the start and end dates of each job episode.

The occupational physician classified job titles and industries according to IndSIC 2015 were blind to the patient’s grouping as a case or as a control. Subjects who worked in more than one of IndSIC’s sections were grouped into the section that reflected the longest period of work.

### INDONESIAN STANDARD OF INDUSTRIAL CLASSIFICATION (INDSIC) 2015 VERSION

The original version of ISIC was developed in 1948, and since that time, the majority of countries around the world have used ISIC as their national activity classification or have developed a national classification derived from ISIC [22]. The latest version of ISIC is ISIC revision four (ISIC rev.4), which comprises of 21 sections, 88 divisions, 238 groups, and 419 classes [8].

The “Indonesian Standard of Industrial Classification” IndSIC was developed by the Centre of Statistical Bureau, or Badan Pusat Statistik (BPS) of Indonesia, which was derived from the ISIC rev. 4. This study used the IndSIC 2015 version which consists of 21 sections (A to U), 88 divisions, 240 groups, 520 subgroups, and 1573 classes. Each item is coded as one letter plus five digits. For example, the code A 01111 can be interpreted as follows: A represents Section A (agriculture, forestry, and fishing), A 01 indicates Division A 01 (crop and animal production, hunting and related service activities), A 011 indicates Group A 011 (crowing of non-perennial crops), A 0111 indicates subgroups A 0111 (growing of cereals (except rice), leguminous crops and oil seeds), and A 01111 indicates Class A 01111 (corn farming) [15].

Among the 21 sections of IndSIC, some sections include a variety of work processes, materials, and substances among their divisions or groups; whereas, in some other sections, they are almost similar. For example, Section C (manufacturing) has 33 divisions such as cement, plastic, chemical, and food industries that are very different when it comes to work processes and exposures, while Section F (construction) has only three nearly similar divisions. These differences can lead to different lung cancer risks among workers in the different divisions of the manufacturing section [16].

### STATISTICAL ANALYSIS

The differences in the proportions of demographic characteristics between cases and controls were evaluated using the chi-square test. We calculated the odds ratio (OR) to investigate the association between different occupational sections of IndSIC 2015 and lung cancer. To control the confounding factors, a multivariate unconditional logistic regression was performed to obtain odds ratio estimates together with 95% confidence intervals (CI) that were adjusted for gender, age, level of education, and smoking habits.

We employed three analysis models to verify the appropriateness of assumptions made in this study and to validate study findings. “Model 1” analysis discovered the ORs among all IndSIC sections using “housewife” as “Reference 1” and Section S (Other services as member of organization, repair of household) as “Reference 2”. Housewives, the “Reference 1”, were considered to have the most similar tasks in all settings, less probability of contact to workplace exposures, the majority of them being non-smokers, and them being at a lower risk of lung cancer compared to other occupations [24,25,26,27,28]. The limitation of this reference group is that they were not part of IndSIC and that they were all females. Section S (Other services as member of organization, repair of household) was chosen to be “Reference 2” because it had the most similar OR to “Reference 1”.

“Model 2” analysis was developed to discover the possible hidden risk of lung cancers in some divisions or groups. We subdivided Sections A (Agriculture, forestry and fishing), B (Mining and quarrying), C (Manufacturing), G (Wholesale and retail trade; repair of motor vehicles and motorcycle), H (Transportation and storage), and Q (Human health and social work activities) into divisions or groups. The remaining sections were not redivided into divisions because most of their divisions are not so different in terms of workplace exposure or work processes. The new classification consisted of the combinations of the sections and divisions or groups. The investigation of ORs was similar to the “Model 1” analysis.

Following the “Model 1” and “Model 2” analysis, sections with significantly increased lung cancer odds ratios were compared to other sections in a third model (“Model 3”). A one-way ANOVA was conducted to compare the mean of ORs among the three models of analyses.

We calculated the population attributable fraction (PAF) for any section or division that had significant OR to estimate the proportion of lung cancer cases that would be prevented if the risk factor was eliminated [23]. Our estimate was made by using the formula, PAF = P(EC) × (OR-1)/OR where OR is the adjusted odds ratio and P(EC) is the proportion of exposed cases [6]. All test decisions were performed at a significance level of 5%. IBM SPSS Statistic for Windows, version 25.0 (IBM Corp., Armonk, NY, USA) was used to analyze the data.

## RESULTS

For 17 months between May 2018 and August 2019, 710 subjects were interviewed, of which 340 subjects were eligible for cases and 370 were eligible for controls. The mean age for lung cancer patients was 58.1 (10.23) years, and the mean age for the controls was 54.5 (10.23) years. The proportion of male smokers who had a smoking history of more than ten pack-years and of workers who had worked for more than ten years was higher in the cases than in the controls. Among the cases, adenocarcinoma dominated the histological cell type (55.9%), and, among controls, tuberculosis (54.6%) was the most prominent diagnosis (***Table 1***).

**Table 1:** Characteristics of subjects.


	CASES (N = 340)	CONTROLS (N = 370)	P VALUE*
	NO. OF SUBJECTS (%)	NO. OF SUBJECTS (%)	

Age			

Mean (SD)	58.1 (10.23)	54.8 (10.23)	0.00

Age categories			

<45 years	35 (10.3)	82 (22.2)	0.00

45–55 years	97 (28.5)	106 (28.6)	

56–65 years	126 (37.1)	117 (31.6)	

66–75 years	68 (20)	52 (14.1)	

>75 years	14 (4.1)	13 (3.5)	

Gender			

Female	128 (37.8)	164 (44.3)	0.08

Male	212 (62.2)	206 (55.7)	

Duration of work			

<10 years	49 (14.4)	87 (23.5)	0.003

10–30 years	156 (45.9)	168 (45.4)	

>30 years	135 (39.7)	115 (31.1)	

Education			

Illiterate	10 (2.9)	10 (2.7)	0.09

Elementary	77 (22.6)	67 (18.1)	

Junior High School	32 (9.4)	40 (10.8)	

Senior High School	132 (38.8)	177 (47.8)	

Bachelor	78 (22.9)	71 (19.2)	

Postgraduate	11 (3.2)	5 (1.4)	

Smoking			

0 to 10 pack-years	174 (51.2)	240 (64.9)	0.001

>10 to 40 pack-years	107 (31.5)	85 (23.0)	

>40 pack-years	59 (17.4)	45 (12.2)	

Histological cell type			

Adenocarcinoma	190 (55.9)		

Large cell	16 (4.7)		

Squamous cell	73 (21.5)		

Unidentified	3 (0.9)		

Small cell	13 (3.8)		

Others	45 (13.2)		

Diagnoses of controls			

No abnormality		30 (8.1)	

Tuberculosis and other lung infections		202 (54.6)	

Chronic lung diseases		66 (17.8)	

Mediastinal mass and other malignancies		40 (10.8)	

Other diseases		32 (8.6)	


* The t test was used for continues variables and χ^2^ test for categorical variables.

In total, 1,095 work histories were collected. ***Table 2*** shows the distribution of subjects for each section of IndSIC, housewife, and unemployment, all of which were stratified by gender. The highest proportion of all subjects was working in Section G (wholesale and retail trade; repair of motor vehicles and motorcycle), followed by Section C (manufacturing). Among female subjects, the highest proportion was in housewives. In almost all sections of IndSIC 2015, the proportion of males was dominant except for Section C (manufacturing), Section K (financial and insurance activities), Section P (education), and Section T (activities of a household as employers; undifferentiated goods and services-producing activities of households for own use).

**Table 2:** Distribution of gender in each section of Indonesian Standard of Industrial Classification 2015.


INDSIC 2015	FEMALE (292)	MALE (418)	TOTAL (710)
	N (%)	N (%)	N (%)

Housewife	83 (28.4)	0 (0)	83 (11.7)

Unemployed	3 (1.0)	2 (0.5)	5 (0.7)

A: Agriculture, forestry and fishing	6 (2.1)	23 (5.5)	29 (4.1)

B: Mining and quarrying	1 (1.3)	8 (1.9)	9 (1.3)

C: Manufacturing	47 (16.1)	47 (11.3)	94 (13.2)

D: Electricity, gas, steam and air conditioning supply	0 (0)	5 (1.2)	5 (0.7)

E: Water supply; sewage, waste management, material recovery	0 (0)	3 (0.7)	3 (0.4)

F: Construction	5 (1.7)	50 (12.0)	55 (7.7)

G: Wholesale and retail trade; repair of motor vehicles and motorcycle	48 (16.4)	91 (21.8)	139 (19.6)

H: Transportation and storage	2 (0.7)	64 (15.3)	67 (9.4)

I: Accommodation and food service activity	6 (2.1)	11 (2.6)	17 (2.4)

J: Information and communication	5 (1.7)	6 (1.4)	11 (1.5)

K: Financial and insurance activities	12 (4.1)	8 (1.9)	20 (2.8)

L: Real estate activities	3 (1.0)	0 (0)	3 (0.4)

M: Professional, scientific and technical activities	2 (0.7)	4 (1.0))	6 (0.8)

O: Public administration and defense; compulsory social security	10 (3.4)	35 (8.4)	45 (6.3)

P: Education	17 (5.8)	18 (4.3)	35 (4.9)

Q: Human health and social work activities	15 (5.1)	8 (1.9)	23 (3.2)

R: Arts, sports and recreation related services	2 (0.7)	7 (1.7)	9 (1.3)

S: Membership organization, repair and other personal services	13 (4.5)	27 (6.5)	40 (5.6)

T: Activities of household as employers; undifferentiated goods-and services-producing activities of households for own use	12 (4.1)	0 (0)	12 (1.6)


“Model 1” analysis found that, compared to “Reference 1”, the adjusted OR for workers in Section A (agriculture, forestry, and fishing) was 3.8 (95% CI = 1.42–10.6), and Section F (Construction) was 2.9 (95% CI = 1.27–6.54). In comparison to “Reference 2”, the adjusted OR for workers in Section A (Agriculture, forestry, and fishing) was 3.6 (95% CI = 1.20–10.43), and Section F (Construction) was 2.6 (95% CI = 1.06–6.20) (***Table 3***).

**Table 3:** Adjusted odds ratio of the association between occupational backgrounds in sections of the Indonesian Standard of Industrial Classification 2015 and lung cancer.


INDSIC 2015	CASES (340)	CONTROLS (370)	ADJUSTED OR (95%CI)^#^	Adjusted OR (95% CI)^##^
	N (%)	N (%)		

Housewife	31 (9.1)	52 (14.1)	Reference	Not included

Unemployed	1 (0.3)	4 (1.1)	0.6 (0.05–5.81)	Not included

A: Agriculture, forestry and fishing	21 (6.2)	8 (2.2)	3.8 (1.42–10.6) *	3.6 (1.20–10.43) *

B: Mining and quarrying	5 (1.5)	4 (1.1)	3.7 (0.82–16.90)	2.6 (0.56–11.95)

C: Manufacturing	42 (12.4)	52 (14.1)	2.8 (0.64–12.86)	1.7 (0.76–3.70)

D: Electricity, gas, steam and air conditioning supply	2 (0.6)	3 (0.8)	1.9 (0.96–3.66)	1.1 (0.16–7.93)

E: Water supply; sewage, waste management, material recovery	2 (0.6)	1 (0.3)	4.2 (0.33–54.53)	3.8 (0.29–48.75)

F: Construction	35 (10.3)	20 (5.4)	2.9 (1.27–6.54) *	2.6 (1.06–6.20) *

G: Wholesale and retail trade; repair of motor vehicles and motorcycle	61 (17.9)	79 (21.4)	1.4 (0.75–2.67)	1.3 (0.60–2.67)

H: Transportation and storage	32 (9.4)	33 (8.9)	1.7 (0.75–3.78)	1.5 (0.65–3.44)

I: Accommodation and food service activity	7 (2.1)	10 (2.7)	1.2 (0.39–3.82)	1.1 (0.33–3.59)

J: Information and communication	4 (1.2)	7 (1.9)	1.3 (0.33–5.16)	1.2 (0.28–4.93)

K: Financial and insurance activities	12 (3.5)	8 (2.2)	2.8 (0.98–8.28)	2.6 (0.82–8.10)

L: Real estate activities	0 (0)	3 (0.8)	0	0

M: Professional, scientific and technical activities	2 (0.6)	4 (1.1)	0.5 (0.09–4.02)	0.5 (0.08–3.83)

O: Public administration and defense; compulsory social security	25 (7.4)	21 (5.7)	1.7 (0.71–3.95)	1.5 (0.61–3.86)

P: Education	17 (5.0)	18 (4.9)	1.3 (0.53–3.43)	1.2 (0.44–3.40)

Q: Human health and social work activities	15 (4.4)	8 (2.2)	2.8 (0.89–8.5)	2.8 (0.89–8.51)

R: Arts, sports and recreation related services	5 (1.5)	4 (1.1)	2.5 (0.58–11.13)	2.3 (0.51–10.35)

S: Membership organization, repair computer and household, and other personal services	15 (4.4)	25 (6.8)	1.1 (0.48–2.62)	Reference

T: Activities of household as employers; undifferentiated goods-and services-producing activities of households for own use	6 (1.8)	6 (1.6)	1.8 (0.53–6.51)	1.7 (0.41–6.55)


The OR was calculated using logistic regression adjusted for age, gender, education, and smoking. ^#^ Reference group: “Housewife”. ^##^ Reference group: Section S (Membership organization, repair computer and household, and other personal services) *Statistically significant, i.e. p ≤ 0.05

When several sections were subdivided into divisions or groups in “Model 2”, subjects who had worked in Division A01 (crop, animal production, and hunting) (OR = 3.9, 95% CI = 1.36–11.25), Division C20 (chemical and chemical product) (OR = 4.8, 95% CI = 1.09–21.60), and Section F (construction) (OR = 2.8, 95% CI = 1.20–6.37) had a significantly higher chance of developing lung cancer compared to “Reference 1”. Division A 02 and 03 (forestry and fishing) of Section A did not show an increased chance of developing lung cancer. Compared to “Reference 2”, subjects in Division A01 (crop, animal production, and hunting) (OR = 3.7, 95% CI = 1.20–11.32) and Section F (construction) (OR = 2.6, 95% CI = 1.07–6.14) maintained a higher chance of developing lung cancer, while subjects working in Division C20 (chemical and chemical product) did not show a statistically significant odds ratio (***Table 4***).

**Table 4:** Adjusted odds ratios of the association between occupational backgrounds classified in sections, divisions, or groups based on the Indonesian Standard of Industrial Classification 2015 and lung cancer.


INDSIC 2015	CASES (340)	CONTROLS (370)	ADJUSTED OR (95%CI)^#^	Adjusted OR (95% CI)^##^
	N (%)	N (%)		

Housewife	31 (9.1)	52 (14.1)	Reference	Not included

Unemployed	1 (0.3)	4 (1.1)	0.6 (0.05–5.86)	Not included

A: Agriculture, forestry and fishing				

A 01: Crop, animal production and hunting	19 (5.6)	7 (1.9)	3.9 (1.36–11.25)*	3.7 (1.20–11.31)*

A 02 & 03: Forestry and fishing	2 (0.6)	1 (0.3)	2.8 (0.22–35.52)	2.5 (0.20–32.35)

B: Mining and quarrying				

B 610: Oil and gas mining	4 (1.2)	(0.0)	~	~

B 510: Coal and lignite mining	1 (0.3)	4 (1.1)	0.5 (0.05–4.80)	0.4 (0.04–4.53)

C: Manufacturing				

C 10: Food industry	5 (1.4)	5 (1.4)	2.5 (0.63–9.80)	2.3 (0.54–9.65)

C 14: Manufacture of wearing apparel	12 (3.5)	17 (4.6)	1.6 (0.67–4.06)	1.5 (0.54–4.10)

C 15: Industry of leather, synthetic, footwear	1 (0.3)	4 (1.2)	0.7 (0.07–6.98)	0.7 (0.07–6.58)

C 17: Industry of pulp, paper, paper board	4 (1.2)	0 (0.0)	~	~

C 20: Chemical and chemical product	7 (2.1)	3 (0.8)	4.8 (1.09–21.60) *	4.5 (0.96 (20.73)

C 21: Pharmaceuticals, medicinal chemical and botanical products	3 (0.9)	0 (0.0)	~	~

C 23: Non-metal mining goods	3 (0.9	4 (1.2)	1.7 (0.32–8.50)	1.6 (0.29–8.39)

C 24: Industry of iron and steel	4 (1.2)	5 (1.4)	1.8 (0.41–8.30)	1.7 (0.37–7.81)

C 26: Computer, electronic and optic industry, semiconductor, and other electronic components	1 (0.3)	4 (1.2)	0.5 (0.05–4.52)	0.4 (0.04–4.36)

C 2910: Four Wheels vehicle industry	4 (1.2)	0 (0)	0	0

C 31: Furniture industry	1 (0.3)	3 (0.8)	0.6 (0.06–6.80)	0.6 (0.05–0.38)

C 32: Other industries	1 (0.3)	3 (0.8)	0.9 (0.09–9.73)	0.8 (0.07-–9.02)

D: Electricity, gas, steam and AC supply	2 (0.6)	3 (0.8)	1.2 (0.18–8.68)	1.1 (0.16–7.82)

E: Water supply; sewage, waste management, material recovery	2 (0.6)	1 (0.3)	4.2 (0.34–53.69)	3.8 (0.29–49.51)

F: Construction	35 (10.3)	20 (5.4)	2.8 (1.20–6.37) *	2.6 (1.07–6.14) *

G: Wholesale and retail trade; repair of motor vehicles and motorcycle				

G 45: Repair of motor vehicles	9 (2.6)	9 (2.4)	1.8 (0.57–5.52)	1.6 (0.51–5.23)

G 47: Retail trade	52 (15.3)	70 (18.9)	1.3 (0.69–2.54)	1.2 (0.57–2.59)

H: Transportation and storage				

H 49: Railroad transportation	25 (7.4)	23 (6.2)	1.8 (0.75–4.23)	1.6 (0.66–3.94)

H 501: Sea and air transport and warehouse, transportation support	7 (2.1)	10 (2.7)	1.3 (0.39–4.20)	1.1 (0.34–3.83)

I: Accommodation and food service activity	7 (2.1)	10 (2.7)	1.2 (0.37–4.01)	1.1 (0.33–3.56)

J: Information and communication	4 (1.2)	7 (1.9)	1.3 (0.32–5.07)	1.2 (0.28–4.95)

K: Financial and insurance activities	12 (3.5)	8 (2.2)	2.8 (0.95–8.13)	2.5 (0.81–8.11)

L: Real estate activities	0 (0)	3 (0.8)	0	0

M: Professional, scientific and technical activities	2 (0.6)	4 (1.2)	0.6 (0.08–3.86)	0.5 (0.07–3.75)

O: Public administration and defense; compulsory social security	25 (7.4)	21 (5.7)	1.6 (0.66–3.78)	1.5 (0.59–3.78)

P: Education	17 (5.0)	18 (4.9)	1.3 (0.50–3.36)	1.2 (0.43–3.39)

Q: Human health and social work activities				

Q 861: Hospital and medical activities	11 (3.2)	6 (1.6)	2.7 (0.83–8.68)	2.5 (0.72–8.67)

Q 869: Nonhospital health-related activities	4 (1.2)	2 (0.6)	3.9 (0.64–23.5)	3.5 (0.54–22.85)

R: Arts, sports and recreation related services	5 (1.5)	4 (1.2)	2.5 (0.56–10.9)	2.3 (0.50–10.38)

S: Membership organization, repair and other personal services	15 (4.4)	25 (6.8)	1.1 (0.46–2.59)	Reference

T: Activities of household as employers; undifferentiated goods-and services-producing activities of households for own use	6 (1.8)	6 (1.6)	1.9 (0.54–6.70)	1.7 (0.44–6.92)


The OR was calculated using logistic regression *adjusted for age, gender, education, and smoking habits. ~ is infinite. # Reference group: “Housewife”. ## Reference group: Section S (Membership organization, repair computer and household, and other personal services). * Statistically significant, i.e. p ≤ 0.05.

***Table 5*** shows the association between occupational backgrounds in Division A01, Division C20, Section F, and the remaining sections of IndSIC 2015 and lung cancer (“Model 3”). The chance of workers developing lung cancer in Division A01 (crop, animal production, and hunting) (OR = 2.7, 95% CI = 1.05–6.76) and Section F (construction) (OR = 1.9, 95% CI = 1.03–3.40) was consistently higher compared to workers of the remaining sections.

**Table 5:** Odds ratio and adjusted odds ratio of the association between lung cancer and occupational backgrounds in Division A01, Division C20, and Section F of the Indonesian Standard of Industrial Classification 2015.


INDSIC 2015	CASES (308)	CONTROLS (314)	ADJUSTED OR (95% CI)
	N (%)	N (%)	

Reference	236 (76.6)	278 (88.5)	1

A 01: Crop, animal production and hunting	19 (6.2)	7 (2.2)	2.7 (1.05–6.76)*

F: Construction	35 (11.4)	20 (6.4)	1.9 (1.03–3.40)*

C 20: Chemical and chemical products	7 (2.3)	3 (1.0)	3.2 (0.79–12.79)


The OR was calculated using logistic regression adjusted for age, gender, education, and smoking. * Statistically significant, i.e. p ≤ 0.05.

The ANOVA showed that there was no statistically significant difference of the mean of ORs among the three models (F (4,103) = 0.44, *p* = 0.78). The PAF for workers in Division A 01 (crop, animal production, and hunting) was 3.9% and for workers in Section F (Construction) was 5.4%. Two divisions in Section C (C17: pulp, paper, and paper products and C21: pharmaceuticals, medicinal chemical, and botanical products) and a division in Section B (B610: oil and gas mining) were the only divisions that had cases without controls.

## DISCUSSION

The present study succeeds in bringing evidence of the excess risk of lung cancer for workers that can be classified under the IndSIC’s Division A01 (Crop, animal production and hunting) and Section F (Construction). The PAF for crop, animal, and hunting workers was 3.9% and construction workers was 5.2%, showing that the contribution of occupational carcinogens contributed to the lung cancer burden in Indonesia. Applying the PAF to the 30,023 incident cases of lung cancer in Indonesia in 2018, we estimated that 1,170 cases were attibutable to occupation in crop, animal, and hunting division and 1,561 cases were attributable to occupations in the construction section in 2018 [9]. The increased risk of lung cancer for agriculture and construction workers was similar to a study performed by Baser, et al. in Turkey. They identified the increased risk of lung cancer among agriculture workers (OR = 1.89, 95% Cl = 1.17–2.98) and workers exposed to inorganic dust (ceramic and pottery workers, construction, and mining) (OR = 1.81, 95% Cl = 1.0–3.25) compared to office workers. They also discovered that the elevated risk for agriculture workers was associated with pesticide use [29]. The odds ratio in our study was slightly higher than in the Baser study but comparable with a cohort study by Alavanja et al [30]. Many other studies have proved that pesticides are associated with an increased risk of lung cancer in agriculture workers [30,31,32,33,34].

Tse et al., after applying the ISIC rev.4 to investigate the risk of lung cancer in Chinese workers, reported that construction workers have a significantly increased risk of having lung cancer compared to workers from other sections (OR = 1.37, 95% CI: 1.01–1.89). Tse et al. further identified that occupational carcinogens associated with the development of lung cancer were caused by silica dust (1.75, 95% CI: 1.16–2.62), welding fumes (1.74, 95% CI: 1.13–2.68), diesel exhaust (2.18, 95% CI: 1.23–3.84), and man-made mineral fibers (7.45, 95% CI: 1.63–34.00) [3]. In addition to the increased risk of lung cancer, a study in California by Calvert et al. indicated that lung cancer in construction workers was diagnosed at an earlier age, at a more advanced stage, and had significantly lower survival rates by three years compared to non-construction workers. The odds ratio in this study was comparable with Calvert et al.’s findings [17].

Bianco and Demers, in a publication about trends in compensation for occupational cancer in Ontario, Canada, noted that lung cancer was the most frequently compensated occupational cancer, especially in the construction, manufacturing, and mining industries [35]. Occupational lung cancer in Korea, reported by Yeon-Soon Ahn and Kyong Sook Jeong, was primarily associated with manufacturing and construction work and were the most common occupations compensating for lung cancer [19]. Both studies indicated that asbestos was responsible for the elevated risks of lung cancer, especially among construction workers.

It is common knowledge that construction workers face a lack of protection and have a high rate of occupational accidents, especially in developing countries. An inspection report in 2018 revealed that up to 80% of construction projects in Jakarta, the capital of Indonesia, did not have health and safety regulations in place and that there were a lack of trained workers [36]. On the other hand, most agriculture workers are informal workers who do not have enough knowledge and resources to protect themselves. Several studies have reported a high prevalence of chronic pesticide intoxication in farmers [37], and other researchers reported that most agricultural workers in Indonesia have been working without personal protective equipment when using pesticides [38].

Siemiatycki et al. and Loomis et al. listed lung carcinogens (Group A) with the occupations or industries in which the carcinogen substances are found [1,39]. This study only identified the elevated risks of lung cancer for Division A01 (crop, animal production, and hunting) and Section F (construction) and could not discover potential increased risk for other occupations or industries that have occupational carcinogens. However, we can observe possible increased risks coming from Division C17 (pulp, paper, and paper products), Division C21 (pharmaceuticals, medicinal chemical, and botanical products), and Division B (oil and gas). Unfortunately, the number of subjects was not sufficient to obtain enough controls. This meant that those divisions did not have controls, only some cases. Therefore, we could not indicate the OR. We were also careful with the increased OR for chemical and chemical product divisions compared to the housewife as a reference. The increased OR was not significant when the reference was from Section S and the other remaining sections. Further research should be conducted to investigate the risks of lung cancer by having a sufficient number of subjects.

There are some limitations in this study, especially of methodological nature. A major limitation is the explorative character of this case-control study, such as the lack of conducting multiple tests for different subgroups. As there are limited studies and data on the risks of lung cancer in Indonesia available, this explorative research has nonetheless added an important first insight into this public health issue, proving that further research in this area is indispensable. Unmatched subjects, possible misclassification of the subjects’ occupations, and recall bias were other limitations of this study. However, the increased chances for agriculture and construction workers to develop lung cancer found in this study were comparable to other previous studies of other countries and indicated no substantial effect of the limitations. Selection bias was also a concern for the hospital-based case-control study. However, almost all of the proportioning of subjects in each section of IndSIC were comparable with the proportion of Indonesian workers based on IndSIC, and this allowed us to assume that the subjects represented the Indonesian population [20,40]. Moreover, the study’s location was the national referral hospital for respiratory diseases and the most prominent center for lung cancer management in Indonesia. This allowed us to assume that we had obtained a representative sample of the lung cancer patients of Indonesia for our study’s aim.

As far as we know, this is the first study in Indonesia to approach occupational lung cancer through the IndSIC classification system. This approach is most convenient since the country has insufficient data on the effects of exposure. For more detailed information, further investigations, to the level of division or even classes that may identify risk that had not appeared at the section level, should be performed. The approach could be extended further by increasing the number of subjects or directly investigating the occupational agents causing lung cancer by using a job exposure matrix.

## CONCLUSIONS

This study filled in the gap of knowledge by bringing significant evidence of how occupational roles correlate with the development of lung cancer among Indonesian workers. It shows the excess risk of lung cancer among workers in Section F (construction) and Division A01 (crop, animal husbandry, and hunting) which could be an early hint of association of some carcinogens with lung cancer development among Indonesian workers. This study confirms the need for improved policy, monitoring, and control of occupational exposure for primary cancer prevention and workers’ compensation purposes. It is needed to ensure that people with work-related lung cancer are diagnosed. Therefore, more training about workplace exposures risk to workers at high risk and training on diagnosing occupational lung cancer need to be provided to health care professionals. Our study results demand further investigations to unravel the possibility that there are even more risk factors for lung cancer among workers in Indonesia in existence. Moreover, this study succeeded in employing IndSIC 2015 as the proxy of occupational exposure to discover occupational disease which can be applied in future occupational health research in Indonesia.

## References

[1] Loomis D, Guha N, Hall AL, Straif K. Identifying occupational carcinogens: an update from the IARC monographs. Occup Environ Med. 2018; 75(8): 593–603. DOI: 10.1136/oemed-2017-104944 29769352 PMC6204931

[2] Algranti E, Buschinelli JTP, De Capitani EM. Occupational lung cancer. Jornal Brasileiro de Pneumologia. 2010; 36(6): 784–794. DOI: 10.1590/S1806-37132010000600017 21225183

[3] Tse LA, Yu IT, Qiu H, Au JSK, Wang X. Occupational risks and lung cancer burden for Chinese men: A population-based case-referent study. Cancer Causes Control. 2012; 23(1): 121–131. DOI: 10.1007/s10552-011-9861-1 22037909

[4] Bovio N, Richardson DB, Guseva Canu I. Sex-specific risks and trends in lung cancer mortality across occupations and economic activities in Switzerland (1990–2014). Occup Environ Med. 2020; 77(8): 540–548. DOI: 10.1136/oemed-2019-106356 32371421

[5] Matrat M, Guida F, Cénée S, et al. Occupational exposure to diesel motor exhaust and lung cancer: A dose-response relationship hidden by asbestos exposure adjustment? The ICARE study. J Cancer Epidemiol. 2015; 2015. DOI: 10.1155/2015/879302 PMC4573883 26425123

[6] Consonni D, De Matteis S, Lubin JH, et al. Lung cancer and occupation in a population-based case-control study. Am J Epidemiol. 2010; 171(3): 323–333. DOI: 10.1093/aje/kwp391 20047975 PMC2808498

[7] Hämäläinen P, Takala J, Kiat TB. Global estimates of occupational accidents and work-related illnesses. 2017. Published online September 2017.

[8] Bray F, Ferlay J, Soerjomataram I, Siegel RL, Torre LA, Jemal A. Global cancer statistics 2018: GLOBOCAN estimates of incidence and mortality worldwide for 36 cancers in 185 countries. CA: A Cancer Journal for Clinicians. 2018; 68(6): 394–424. DOI: 10.3322/caac.21492 30207593

[9] IACR – GLOBOCAN. http://www.iacr.com.fr/index.php?option=com_content&view=article&id=101&Itemid=578. Accessed June 1, 2019.

[10] Mahendradhata Y, Trisnantoro L, Listyadewi S, Soewondo P, Marthias T, et al. The Republic of Indonesia Health System Review. WHO Regional Office for South-East Asia; 2017. https://apps.who.int/iris/handle/10665/254716. Accessed November 28, 2020.

[11] Tjindarbumi D, Mangunkusumo R. Cancer in Indonesia, present and future. Japanese Journal of Clinical Oncology. 2002; 32(suppl 1): S17–S21. DOI: 10.1093/jjco/hye123 11959873

[12] BPJS Ketenagakerjaan. https://www.bpjsketenagakerjaan.go.id/. Accessed October 13, 2019.

[13] International Standard Industrial Classification of All Economic Activities (ISIC). ILOSTAT. https://ilostat.ilo.org/resources/methods/classification-economic-activities/. Accessed March 27, 2020.

[14] Bantuan dan Tutorial | SPK Online – Sistem Pencarian Kode Klasifikasi Online. https://spkonline.bps.go.id/spkonline/help/klasifikasi/2. Accessed November 30, 2020.

[15] 20151123 KBLI 2015 Merge.pdf – KBLI-2015.pdf. http://www2.bkpm.go.id/images/uploads/prosedur_investasi/file_upload/KBLI-2015.pdf. Accessed August 5, 2017.

[16] Mannetje A ‘t, Kromhout H. The use of occupation and industry classifications in general population studies. Int J Epidemiol. 2003; 32(3): 419–428. DOI: 10.1093/ije/dyg080 12777430

[17] Calvert GM, Luckhaupt S, Lee S-J, et al. Lung cancer risk among construction workers in California, 1988–2007. Am J Ind Med. 2012; 55(5): 412–422. DOI: 10.1002/ajim.22010 22237930

[18] Suraya A, Nowak D, Sulistomo AW, et al. Asbestos-related lung cancer: A hospital-based case-control study in Indonesia. International Journal of Environmental Research and Public Health. 2020; 17(2): 591 DOI: 10.3390/ijerph17020591 PMC7013975 31963324

[19] Ahn Y-S, Jeong KS. Epidemiologic characteristics of compensated occupational lung cancers among Korean workers. Journal of Korean Medical Science. 2014; 29(11): 1473–1481. DOI: 10.3346/jkms.2014.29.11.1473 25408577 PMC4234913

[20] Sicca SP. BPS: Jumlah penduduk bekerja triwulan I 2018 sebanyak 127,07 juta. tirto.id. https://tirto.id/bps-jumlah-penduduk-bekerja-triwulan-i-2018-sebanyak-12707-juta-cJ5D. Accessed June 2, 2020.

[21] Tsim S, Kelly C, Alexander L, et al. Diagnostic and prognostic biomarkers in the rational assessment of mesothelioma (DIAPHRAGM) study: protocol of a prospective, multicentre, observational study. BMJ Open. 2016; 6(11): e013324 DOI: 10.1136/bmjopen-2016-013324 PMC5168514 27884852

[22] United Nations, ed. International Standard Industrial Classification of All Economic Activities (ISIC). Rev. 4. United Nations; 2008.

[23] Rezende LFM de, Eluf-Neto J, Rezende LFM de, Eluf-Neto J. Population attributable fraction: planning of diseases prevention actions in Brazil. Revista de Saúde Pública. 2016; 50 DOI: 10.1590/S1518-8787.2016050006269 27305404 PMC4902656

[24] Quinn MM, Smith PM. Gender, work, and health. Ann Work Expo Health. 2018; 62(4): 389–392. DOI: 10.1093/annweh/wxy019 29617721

[25] Scarselli A, Corfiati M, Di Marzio D, Marinaccio A, Iavicoli S. Gender differences in occupational exposure to carcinogens among Italian workers. BMC Public Health. 2018; 18 DOI: 10.1186/s12889-018-5332-x PMC5870210 29587708

[26] Sutandyo N, Suratman E. Non-small cell lung carcinoma in women: A retrospective cohort study in Indonesia. Acta Med Indones. 2018; 50(4): 291–298. 30630993

[27] Robinson CF, Sullivan PA, Li J, Walker JT. Occupational lung cancer in US women, 1984–1998. Am J Ind Med. 2011; 54(2): 102–117. DOI: 10.1002/ajim.20905 21259296

[28] Joshi SK, Bråtveit M, Moen B. Possible occupational lung cancer in Nepal. JNMA; Journal of the Nepal Medical Association. 2003; 42: 1–5. DOI: 10.31729/jnma.659

[29] Baser S, Duzce O, Evyapan F, Akdag B, Ozkurt S, Kiter G. Occupational exposure and thoracic malignancies, is there a relationship? J Occup Health. 2013; 55(4): 301–306. DOI: 10.1539/joh.13-0097-FS 23796595

[30] Alavanja MCR, Dosemeci M, Samanic C, et al. Pesticides and lung cancer risk in the agricultural health study cohort. Am J Epidemiol. 2004; 160(9): 876–885. DOI: 10.1093/aje/kwh290 15496540

[31] Barthel E. Increased risk of lung cancer in pesticide-exposed male agricultural workers. Journal of Toxicology and Environmental Health. 1981; 8(5–6): 1027–1040. DOI: 10.1080/15287398109530135 7338938

[32] Bonner MR, Freeman LEB, Hoppin JA, et al. Occupational exposure to pesticides and the incidence of lung cancer in the agricultural health study. Environ Health Perspect. 2017; 125(4): 544–551. DOI: 10.1289/EHP456 27384818 PMC5381995

[33] Weichenthal S, Moase C, Chan P. A review of pesticide exposure and cancer incidence in the agricultural health study cohort. Ciência & Saúde Coletiva. 2012; 17(1): 255–270. DOI: 10.1590/S1413-81232012000100028 22218559

[34] Boulanger M, Tual S, Lemarchand C, et al. Lung cancer risk and occupational exposures in crop farming: results from the AGRIculture and CANcer (AGRICAN) cohort. Occup Environ Med. 2018; 75(11): 776–785. DOI: 10.1136/oemed-2017-104976 30185443

[35] Del Bianco A, Demers PA. Trends in compensation for deaths from occupational cancer in Canada: A descriptive study. CMAJ Open. 2013; 1(3): E91–96. DOI: 10.9778/cmajo.20130015 PMC4114060 25077112

[36] Gloal Construction Review. 80% of Jakarta projects unsafe, says official report 2018. http://www.globalconstructionreview.com/news/80-jakarta-projects-unsafe-says-official-report/. Accessed June 18, 2020.

[37] Yuantari MC, Setiani O, Nurjazuli N. Studi ekonomi lingkungan Ppnggunaan pestisida dan dampaknya pada kesehatan petani di area pertanian hortikultura desa sumber rejo kecamatan ngablak kabupaten magelang. Jurnal Kesehatan Lingkungan Indonesia. 2009; 8(2): 63–69. DOI: 10.14710/jkli.8.2.63-69

[38] Yuantari MGC, Widianarko B, Sunoko HR. Analisis risiko pajanan pestisida terhadap kesehatan petani. KEMAS: Jurnal Kesehatan Masyarakat. 2015; 10(2): 239–245. DOI: 10.15294/kemas.v10i2.3387

[39] Siemiatycki J, Richardson L, Straif K, et al. Listing occupational carcinogens. Environmental Health Perspectives. 2004; 112(15): 1447–1459. https://www.jstor.org/stable/3435600 Accessed April 16, 2020. DOI: 10.1289/ehp.7047 15531427 PMC1247606

[40] Badan Pusat Statistik. https://www.bps.go.id/. Accessed August 8, 2017.

